# The nutritional composition and anti-obesity effects of an herbal mixed extract containing *Allium fistulosum* and *Viola mandshurica* in high-fat-diet-induced obese mice

**DOI:** 10.1186/s12906-015-0875-1

**Published:** 2015-10-16

**Authors:** Yoon-Young Sung, Seung-Hyung Kim, Byoung Wan Yoo, Ho Kyoung Kim

**Affiliations:** Mibyeong Research Center, Korea Institute of Oriental Medicine, Yuseong-daero 1672, Yuseong-gu, Daejeon 305-811 Republic of Korea; Institute of Traditional Medicine and Bioscience, Daejeon University, Daejeon, 300-716 Republic of Korea; Institute of JinAn Red Ginseng, Jinan-Eup, Jinan-Gun, Chonbuk 567-801 Republic of Korea

**Keywords:** *Allium fistulosum*, Hyperlipidemia, Mixture, Nutritional, *Viola mandshurica*, Vitamin

## Abstract

**Background:**

In traditional oriental medicine, *A. fistulosum* and *V. mandshurica* are considered to be effective in promoting blood circulation. Therefore, in this study, we investigated whether a solution containing both *A. fistulosum* and *V. mandshurica* (AFE + VME) extracts has synergistic effects on the treatment of hyperlipidemia and obesity.

**Methods:**

Anti-obesity effects of an herbal extract containing *Allium fistulosum* and *Viola mandshurica* (AFE + VME) were investigated in high-fat diet (HFD)-induced obese mice. AFE + VME was orally administrated to mice with the HFD at a dose of 200 mg/kg/day for 8 weeks. We observed the effects of mixed extract on body weight, fat mass, serum lipid levels, and mRNA expression levels of lipid metabolism-related genes in the adipose tissue of mice.

**Results:**

The nutritional analysis revealed that this mixed extract is high in carbohydrate (72.2 g/100 g) and protein (11.5 g/100 g); low in fat (1.7 g/100 g); rich in vitamins E (4.8 mg/100 g), B_1_ (14.8 mg/100 g), B_2_ (1.0 mg/100 g), niacin (7.9 mg/100 g), and folic acid (1.57 mg/100 g); and rich in minerals such as calcium (600 mg/100 g), iron (106.1 mg/100 g), and zinc (5.8 mg/100 g). The oral administration of AFE + VME in obese mice reduced body weight, tissue weight, adipocyte size, and lipid accumulation in the liver compared with HFD control mice. AFE + VME also decreased serum triglyceride, total cholesterol, and leptin concentrations. Furthermore, AFE + VME markedly increased the mRNA expression of peroxisome proliferator-activated receptor-γ (PPAR-γ), uncoupling protein-2 (UCP-2), and adiponectin and decreased leptin expression in the epididymal white adipose tissue. Our results suggest that the extract containing *A. fistulosum* and *V. mandshurica* improved lipid metabolism via the up-regulation of PPAR-γ, UCP-2, and adiponectin expression and the down-regulation of leptin in HFD-induced obese mice.

**Conclusions:**

Therefore, the extract containing *Allium fistulosum* and *Viola mandshurica* may be a potentially effective therapy for obesity and its related metabolic disorders such as hyperlipidemia and insulin resistance.

## Background

*Allium fistulosum* (commonly known as welsh onion or scallions), a perennial plant in the Alliaceae family, is used as a major flavoring agent in Chinese, Japanese, and Korean cuisine. *A. fistulosum* also has been traditionally used to treat common colds, headache, abdominal pain, and cardiovascular disease [1]. Several studies have indicated that *A. fistulosum* has anti-platelet, anti-oxidative, anti-hypertensive, and anti-hyperlipidemic properties [2–4]. *Viola mandshurica* (Commonly known as violet), a perennial herb in the Violaceae family, is a traditional herbal medicine with expectorant, diuretic, and anti-inflammatory effects [5]. It also has been reported that *V. mandshurica* has anti-oxidative and anti-diabetic effects [6]. In traditional oriental medicine, *A. fistulosum* and *V. mandshurica* also are considered to be effective in promoting blood circulation [7, 8].

Obesity is a serious public health issue and plays a critical role in the pathogenesis of hypertension, dyslipidemia, diabetes mellitus, stroke, osteoarthritis, coronary heart disease, certain types of cancer (e.g., colon, breast, gall bladder), and impaired fertility [9]. Due to the potentially hazardous side effects of the anti-obesity drugs such as orlistat and sibutramine that are currently on the market, a wide variety of natural products to treat obesity are under exploration. These products, which include crude extracts and isolated compounds from plants, may be safe and effective alternative strategies for treating obesity in the future [10]. A previous study from our group showed that *A. fistulosum* extract suppressed the increase in body weight, fat mass, and lipid levels in high-fat-diet (HFD)-induced obese mice via the down-regulation of obesity-related lipogenic genes [11]. Another study from our group showed that *V. mandshurica* extract and its primary constituent, esculetin, inhibited adipocyte differentiation in 3 T3-L1 cells, and suppressed body weight, lipogenesis, and epididymal fat accumulation via the activation of AMP-activated protein kinase in HFD-induced obese mice [12]. These observations indicate that *A. fistulosum* and *V. mandshurica* extracts may be a good candidate for the control of obesity and hyperlipidemia [11, 12]. The combination of various herbal extracts may synergistically increase or decrease the activity or toxicity of drugs [13]. Therefore, in this study, we investigated whether a solution containing both *A. fistulosum* and *V. mandshurica* (AFE + VME) extracts has synergistic effects on the treatment of hyperlipidemia and obesity in HFD-induced obese mice.

## Methods

### Extract preparation

*A. fistulosum* and *V. mandshurica* were purchased from Jeongdo Medicinal Herb Co. (Guri, Korea) and authenticated based on their microscopic and macroscopic characteristics by the Classification and Identification Committee of the Korea Institute of Oriental Medicine (KIOM). A mixed extract (200 g) was prepared with a 1:1 ratio of *A. fistulosum* (100 g) and *V. mandshurica* (100 g). The mixing ratio of extract was based on the dosage ratios from previous *in vitro* and *in vivo* studies. The *A. fistulosum* (200 g), *V. mandshurica* (200 g), and mixed herbs (200 g) were boiled in distilled water at 100 °C for 2 h. The extracts then were filtered, lyophilized, and subsequently stored at −20 °C. The yield of the dried extracts from *A. fistulosum* and *V. manshurica* was 11.25 % (w/w) and 15 % (w/w), respectively. The yield of the mixed extract was 11. 25 % (w/w).

### Nutritional content

One hundred grams sample of the mixed extract (AFE + VME) containing *A. fistulosum* and *V. mandshurica* was analyzed for nutritional components by the Jeonnam Biofood Technology Center (Jeonnam, Korea). The extract was analyzed for carbohydrate, protein, fat, cholesterol, vitamins, and minerals. All tests performed were in compliance with the standards recommended by the Association of Analytical Communities/Association of Official Agricultural Chemist (AOAC) and American Association of Cereal Chemists (AACC). The cholesterol and vitamin levels were measured using high-performance liquid chromatography (HPLC 1200 series, Alltech, USA) and the mineral contents were assessed using inductively coupled plasma optical emission spectrometry (ICP-MS XSERIES2, Thermo, USA).

### Animals and diets

Eight-week-old male C57BL/6 J mice were purchased from The Jackson Laboratory (Bar Harbor, ME, USA) and housed in an air-conditioned room at a temperature of 21 ± 2 °C and humidity of 50 ± 5 % under a 12:12 light:dark cycle. The mice were fed a commercial diet and water ad libitum for 1 week. Mice then were fed a high-fat diet (Rodent Diet D12492, Research Diet, New Brunswick, NJ, USA) consisting of 60 % fat, 20 % protein, and 20 % carbohydrate, to induce obesity. Normal control mice were provided a commercial standard diet (AIN-76A, Research Diet, New Brunswick, NJ, USA) consisting of 11.5 % fat, 20.8 % protein, and 67.7 % carbohydrate. Diets were purchased from Orient Bio Inc. (Seongnam, Korea).

The mice were randomly divided into five groups and each of the five groups included 5 mice. The mice were respectively fed a normal diet (ND), a high-fat diet (HFD), a high-fat diet plus *A. fistulosum* extract (AFE), a high-fat diet plus *V. mandshurica* extract (VME), and a high-fat diet plus a 1:1 ratio mixed extract of *A. fistulosum* and *V. mandshurica* (AFE + VME) for 8 weeks. AFE, VME, and the mixed extract were dissolved in normal saline and administrated orally to the mice (200 mg/kg/day). The normal and HFD-control mice were treated with vehicle (normal saline) only. Body weight and food intake were monitored once per week. Obesity is generally defined as being 20 % or more over ideal body weight. After one week of feeding, the body weight of the HFD group was approximately 20 % higher than the normal control group. All animal procedures were performed according to the Guide for the Care and Use of Laboratory Animals of the National Institutes of Health and was approved by the Institutional Animal Care and Use Committee of Daejeon University in Daejeon, Korea (Approval No. DJUARB2013-022). Food efficiency ratio was calculated as follows: Food efficiency ratio (%) = [Body weight gain (g/day) / food intake (g/day)] × 100.

### Serum assays

At the end of the experimental period, the mice were anesthetized with ether following an overnight fast. Blood was drawn from the abdominal aorta into a BD Microtainer Blood collection tube (BD, Plymouth, UK). Whole blood samples were centrifuged at 2000 × g for 15 min at 4 °C, and the separated serum was stored at −70 °C until analysis. The serum biochemical concentrations of total cholesterol, HDL cholesterol, LDL cholesterol, triglycerides, glucose, creatinine, aspartate aminotransferase (AST), and alanine aminotransferase (ALT) were determined using an automatic analyzer (Express Plus, Chiron Diagnostics, East Walpole, MA, USA) with reagents (BioClinical System, Gyeonggi-do, Korea). Serum leptin levels were measured by immunoassay (ELISA) using commercially available kits (Linco Research, Charles, MO, USA). The absorbance was measured using a microplate spectrophotometer (BioRad, Hercules, CA, USA).

### Weight and histology analysis of liver and adipose tissues

After collecting the blood, the liver and white adipose tissues (subcutaneous and epididymal fats) were removed from the mice and weighed immediately. For adipocyte staining, the liver and visceral adipose tissue were fixed in 10 % neutral formalin solution for 1 day, and embedded in paraffin. All tissues were cut with a thickness of 6 μm, and stained with hematoxylin and eosin. To quantify adipocyte size, the stained sections were analyzed using light microscopy (Olympus BX51, Olympus Optical Co., Tokyo, Japan) and an image analysis program (Image-Pro Plus 5.0, Media Cybernetics, Silver Spring, MD, USA).

### Real-time quantitative RT-PCR

The adipose tissue was homogenized and total RNA from epididymal adipose tissue was isolated with TRI reagent (Sigma, St. Louis, MO, USA) and digested with DNase I (Life Technologies, Grand Island, NY, USA) to remove chromosomal DNA. Five μg of total RNA were reverse transcribed into cDNA with the First Strand cDNA Synthesis kit (Amersham Pharmacia, Piscataway, NJ, USA). The real-time quantitative PCR was performed using the Applied Biosystems 7500 Real-Time PCR system (Applied Biosystems, Grand Island, NY, USA) as described. The primer sequences and the probe sequence are shown in Table 1. The probes were labeled with 6-carboxy-fluorescein (FAM), which is a fluorescent reporter dye. The GAPDH probe as endogenous control was purchased from Applied Biosystems (VIC dye-labeled MGB probe, Grand Island, NY, USA). Each cDNA sample that contained all unknown cDNA and an equal amount of GAPDH cDNA was amplified with the TaqMan Universal PCR master mixture containing DNA polymerase according to the manufacturer’s instructions (PE Applied Biosystems, Foster, CA, USA). The PCR conditions were 2 min at 50 °C, 10 min at 95 °C, 15 s at 95 °C, and 1 min at 60 °C for 40 cycles. The concentration of the target gene was determined using the comparative Ct method (i.e., the threshold cycle number at the cross-point between the amplification plot and the threshold) according to the manufacturer’s instructions.

**Table 1:** The primer and probe sequences used in the real-time RT-PCR analysis

Genes	Probe and primer	Sequence	GeneBank accession number
Leptin	Sense	5′-CCAAAACCCTCATCAAGACC-3′	NM_008493
	Antisense	5′-GTCCAACTGTTGAAGAATGTCCC-3′	
UCP-2	Sense	5′-CCGCATTGGCCTCTACGACTCT-3′	NM_011671
	Antisense	5′-CCCCGAAGGCAGAAGTGAAGTG-3′	
Adiponectin	Sense	5′-CCCAAGGGAACTTGTGCAGGTTGGATG-3′	NM_009605
	Antisense	5′-GTTGGTATCATGGTAGAGAAGAAAGCC-3′	
PPAR-γ	FAM	5′-TCGGAATCAGCTCTGTGGACCTCTCC-3′	NM_011146
GAPDH	VIC	5′-TGCATCCTGCACCACCAACTGCTTAG-3′	NM_008084

### Statistical analysis

The data were analyzed by a one-way analysis of variance (ANOVA) followed by Tukey’s range tests using statistical software Origin v.8.6. All data are presented as the mean ± standard error (SE). Significant differences were accepted when the *p*-value was less than 0.05.

## Results

### Nutritional analysis

The nutritional composition of the mixed extract (AFE + VME) is shown in Table 2. The mixed extract contains 22 % of the recommended daily allowance (RDA) of carbohydrate (72.2 g/100 g), 21 % of the RDA of protein (11.5 g/100 g), 3 % of the RDA of fat (1.7 g/100 g), and non-detectable levels of cholesterol. The mixed extract also is rich in vitamins E (4.8 mg/100 g, 48 % of the RDA), B_1_ (14.8 mg/100 g, 1480 % of the RDA), B_2_ (1.0 mg/100 g, 83 % of the RDA), niacin (7.9 mg/100 g, 61 % of the RDA), and folic acid (1.57 mg/100 g, 394 % of the RDA) and minerals such as calcium (86 % of the RDA), iron (884 % of the RDA), and zinc (5.8 mg/100 g, 68 % of the RDA).

**Table 2:** Analysis of nutritional components in the mixed extract

Components	Values (units/100 g)	RDA^a^
Energy (Kcal)	350.1	
Carbohydrate (g)	72.2	330
Protein (g)	11.5	55
Fat (g)	1.7	50
Cholesterol (mg)	0	300
Sodium (mg)	100.6	2000
Vitamin A (μg)	ND^b^	700
Vitamin C (mg)	ND^b^	100
Vitamin E (mg)	4.8	10
Vitamin B_1_ (mg)	14.8	1
Vitamin B_2_ (mg)	1.0	1.2
Niacin (mg)	7.9	13
Vitamin B_6_ (mg)	ND^b^	1.5
Folic acid (mg)	1.57	0.4
Calcium (mg)	600.8	700
Iron (mg)	106.1	12
Zinc (mg)	5.8	8.5

^a^RDA, recommended daily allowance

^b^ND, not detected

### Body weight, fat mass, and food efficiency ratio

**Fig. 1:**
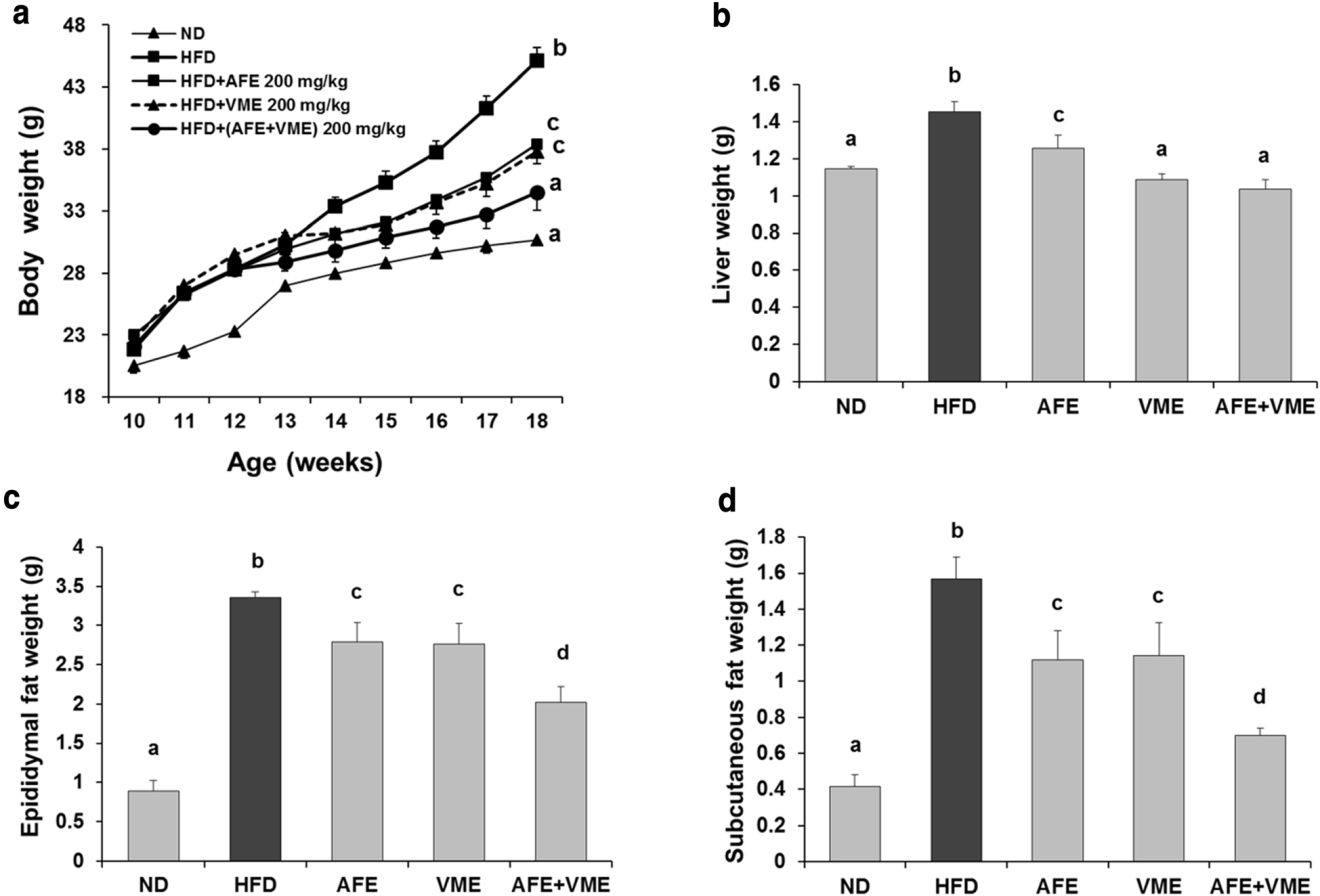
Effect of AFE, VME, and the mixed extract of *Allium fistulosum* and *Viola mandshurica* (AFE + VME) on body weight, liver weight, and epididymal or subcutaneous fat weights in HFD-induced obese mice. **a** Body weight, **b** liver weight, **c** epididymal fat weight, **d** subcutaneous fat weight. Values are presented as the mean ± SE. Values within arrow with different letters are significantly different from each other at *p* <0.05 as determined by Tukey’s range test

The final body weight and body weight gain were significantly lower in mice fed with AFE, VME, or the combination diet (AFE + VME) compared with the HFD control mice, and the largest effect was observed among mice on the combination diet (Fig. 1a and Table 3). Food intake did not differ significantly among the groups (Table 3). The food efficiency ratio was significantly lower in the mice fed AFE, VME, or the combination diet (AFE + VME) compared with the HFD mice (Table 3). The weight of the liver, epididymal fat, and subcutaneous fat was higher in the HFD group compared with the ND group. These tissue weights were significantly decreased in mice fed the AFE, VME, or the combination diet, and the largest effect was observed among mice fed the combination diet (Fig. 1b-d).

**Table 3:** The effects of each diet on body weight, food intake, and the food efficiency ratio in HFD-induced obese mice

	ND	HFD	AFE	VME	(AFE + VME)
Final body weight, g	30.70 ± 0.52^a^	45.10 ± 1.10^b^	38.40 ± 1.72^c^	37.80 ± 0.85^c^	33.75 ± 1.47^a^
Body weight gain, g/day	0.18 ± 0.01^a^	0.38 ± 0.02^b^	0.25 ± 0.03^c^	0.22 ± 0.02^c^	0.17 ± 0.03^a^
Food intake, g/day	5.14 ± 0.00	2.92 ± 0.00	2.84 ± 0.00	2.82 ± 0.00	2.29 ± 0.03
Food efficiency ratio, %	3.57 ± 0.14^a^	13.10 ± 0.57^b^	9.78 ± 0.97^c^	7.80 ± 0.66^c^	7.20 ± 1.01^c^

Food efficiency ratio (%) = body weight gain/food intake × 100. The values are expressed as the mean ± SE (*n* = 5)

Values within arrow with different letters are significantly different from each other at *p* <0.05 as determined by Tukey’s range test

### Serum biochemical analysis

**Fig. 2:**
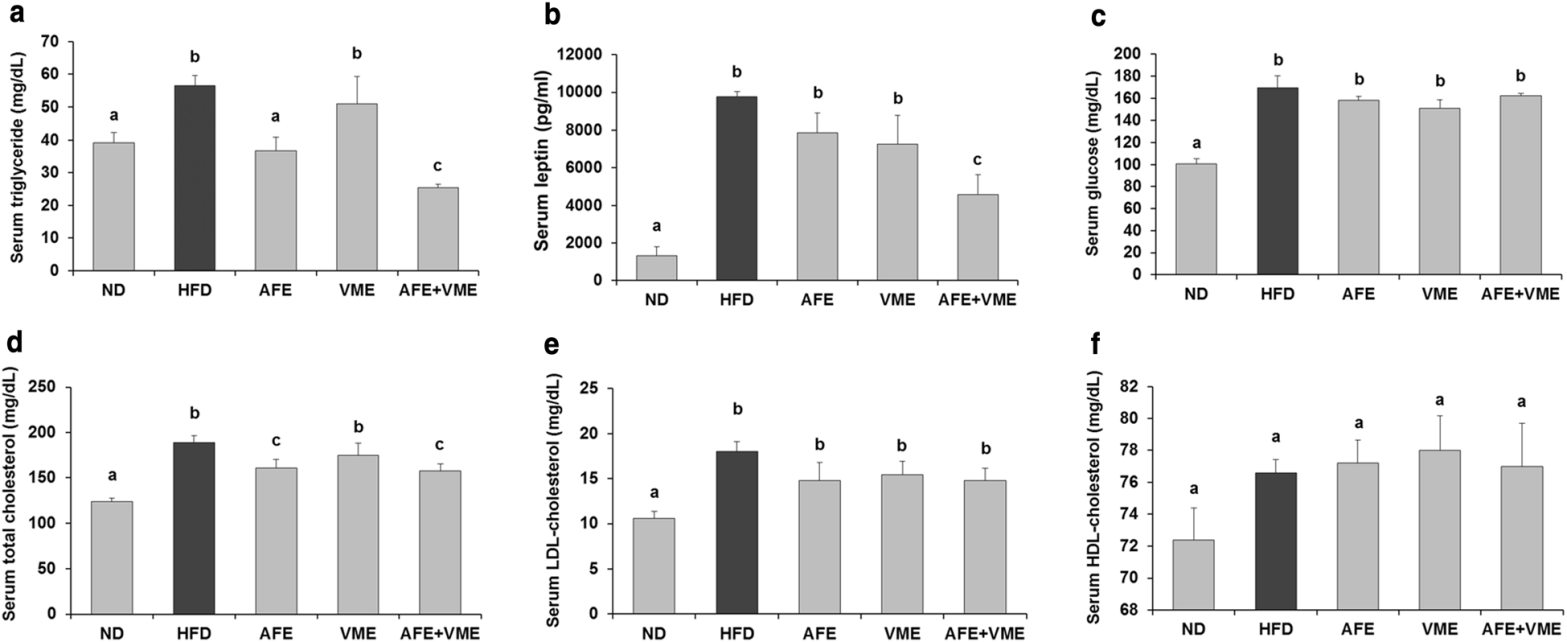
Effect of AFE, VME, and the mixed extract of *Allium fistulosum* and *Viola mandshurica* (AFE + VME) on blood biochemistry parameters in HFD-induced obese mice. Serum **a** triglyceride, **b** leptin, **c** glucose, **d** total cholesterol, **e** LDL-cholesterol, and **f** HDL-cholesterol levels. Values are presented as the mean ± SE. Values within arrow with different letters are significantly different from each other at *p* <0.05 as determined by Tukey’s range test

**Fig. 3:**
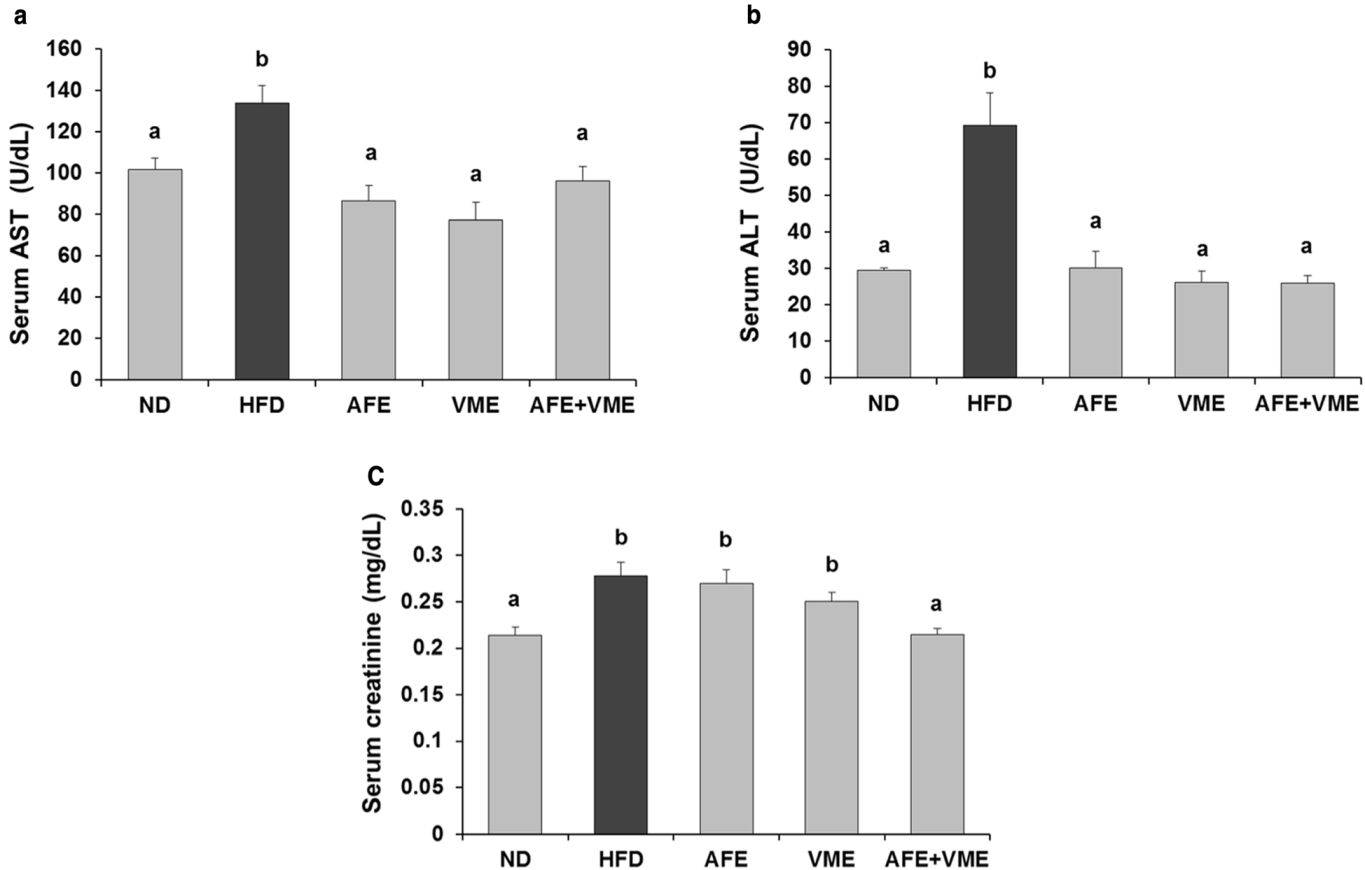
Effect of AFE, VME, and the mixed extract of *Allium fistulosum* and *Viola mandshurica* (AFE + VME) on serum AST, ALT, and creatinine levels in HFD-induced obese mice. Serum **a** AST and **b** ALT, and **c** creatinine levels. Values are presented as the mean ± SE. Values within arrow with different letters are significantly different from each other at *p* <0.05 as determined by Tukey’s range test

The AFE, VME, and AFE + VME diets significantly inhibited the HFD-induced increase in serum triglycerides and total cholesterol levels (Fig. 2), and the combination diet (AFE + VME) was most effective in reducing serum triglyceride, leptin, and total cholesterol levels. The AFE and VME reduced serum leptin levels, but not significant. Serum glucose, LDL cholesterol, and HDL cholesterol levels did not differ among the groups. To evaluate potential toxicity of the mixed extract, we measured serum markers of liver and kidney injuries. AST and ALT were significantly lower in the AFE, VME, and AFE + VME groups, and these levels were similar between the AFE + VME and ND groups (Fig. 3a-b). Serum creatinine level was significantly lower in the AFE + VME group compared with the HFD group, and this level was similar to that in the ND group (Fig. 3c). Therefore, the combination of extracts decreased the toxicity on the liver and kidney of the mice.

### Histological examination of liver and adipose tissue

**Fig. 4:**
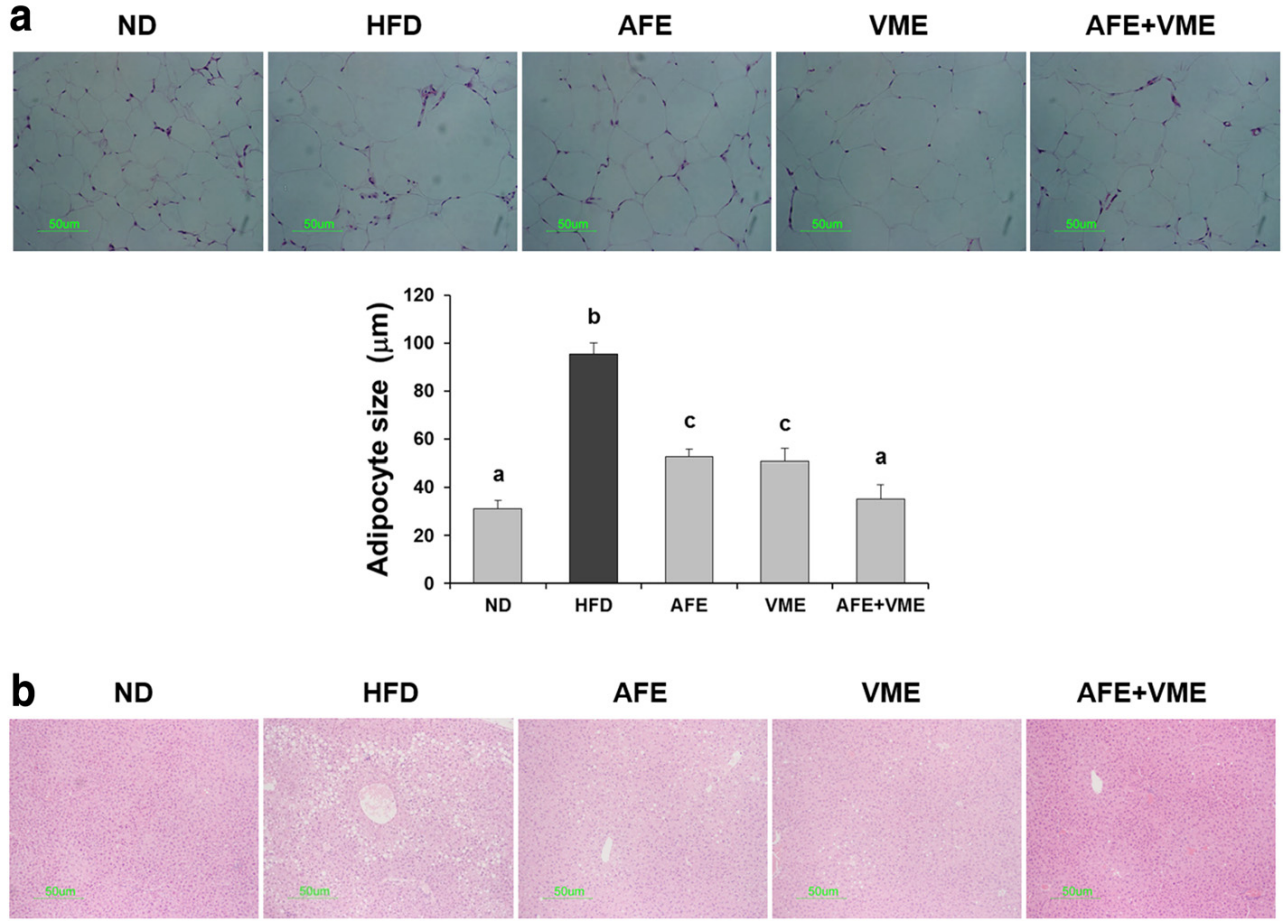
Effect of AFE, VME, and the mixed extract of *Allium fistulosum* and *Viola mandshurica* (AFE + VME) on histology of epididymal adipose tissue and liver in HFD-induced obese mice. Representative photographs of **a** epididymal adipose tissue and **b** liver of hematoxylin and eosin stained sections (magnification, 400×). Values are presented as the mean ± SE. Values are presented as the mean ± SE. Values within arrow with different letters are significantly different from each other at *p* <0.05 as determined by Tukey’s range test

The adipocyte size in the epididymal white adipose tissue was larger in the HFD group compared with the ND group. The adipocyte size was significantly smaller in the AFE, VME, and AFE + VME groups, and the adipocyte size in the AFE + VME group was similar to that in the ND group (Fig. 4a). Fat accumulation in the liver also was lower in the AFE, VME, and AFE + VME (Fig. 4b).

### Gene expression in epididymal white adipose tissue

**Fig. 5:**
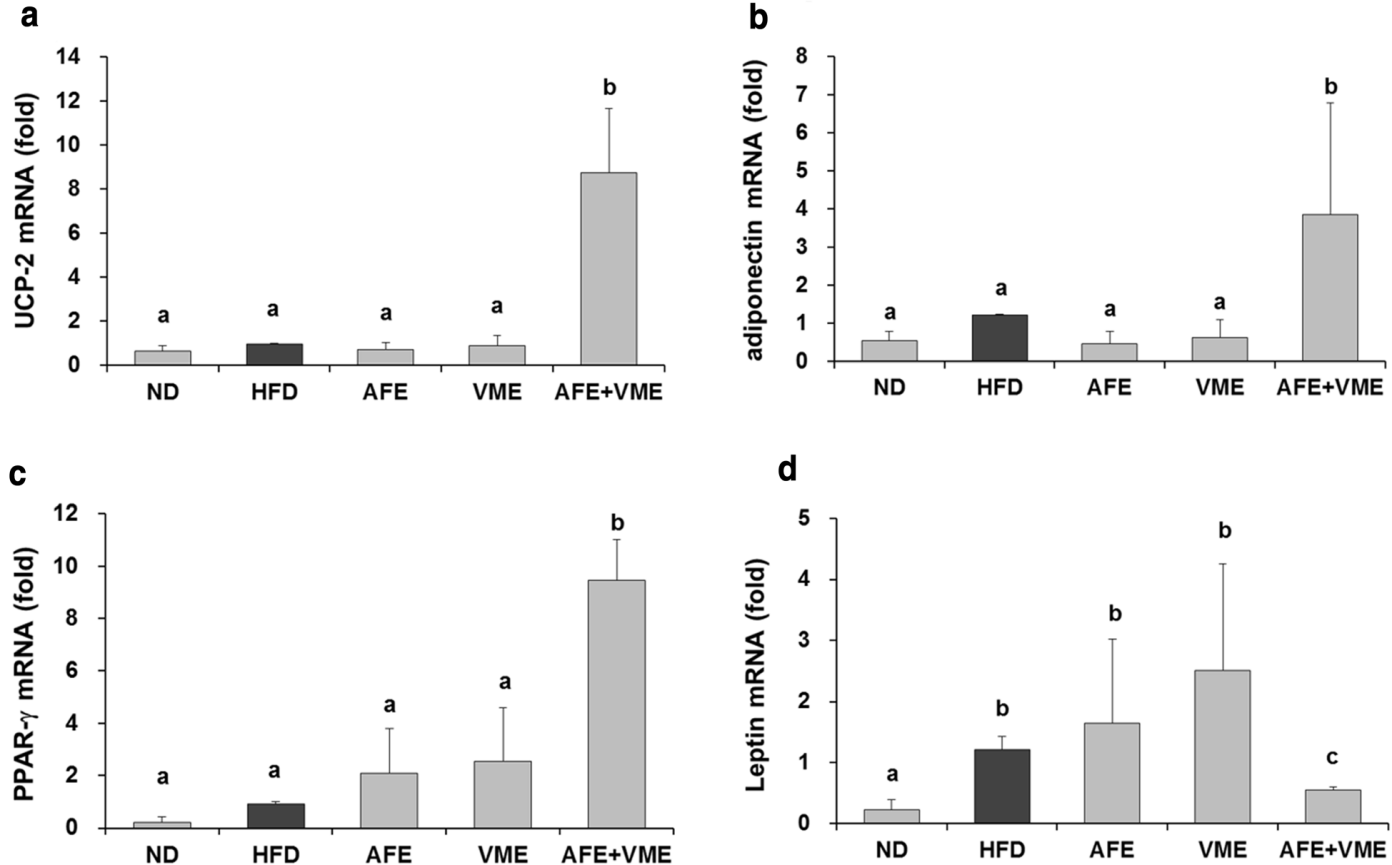
Effect of AFE, VME, and the mixed extract of *Allium fistulosum* and *Viola mandshurica* (AFE + VME) on mRNA levels in the epididymal adipose tissue. **a** UCP-2, **b** adiponetin, **c** PPAR-γ, **d** leptin. Values are presented as the mean ± SE. Values within arrow with different letters are significantly different from each other at *p* <0.05 as determined by Tukey’s range test

The mRNA expression levels of UCP-2, adiponectin, and PPAR-γ were significantly higher in the AFE + VME group compared with the HFD group (Fig. 5a-c). The mRNA levels of leptin were significantly higher in the HFD group compared with the ND group, and were lower in the combination group (AFE + VME) (Fig. 5d). The AFE and VME increased mRNA levels of leptin, but not significant.

## Discussion

The mixed extract of *A. fistulosum* and *V. mandshurica* was rich in vitamins (E, B_1_, B_2_, niacin, and folic acid) and essential minerals (calcium, iron, and zinc). Vitamins and minerals play crucial roles in obesity and obesity-related diseases. Vitamin E has been shown to regulate the expression of adipocytokines such as leptin and adiponectin and decrease oxidative stress in an HFD-induced obese rat [14]. Vitamin B_1_ (thiamine) has been shown to prevent obesity through the alleviation of body weight gain, adipocyte hypertrophy, and liver steatosis [15]. Folic acid supplementation in mice has been shown to have a protective effect against HFD-induced body mass, hepatic oxidative stress, and liver injury [16]. Niacin treatment has been shown to increase adiponectin levels and attenuate obesity-induced adipose tissue inflammation as well as obesity in HFD-fed mice [17]. The mixed extract of *A. fistulosum* and *V. mandshurica* also included high levels of iron (884 % of the RDA). HFD-induced obesity leads to reduced iron storage, and a grape seed and skin extract has been shown to prevent dyslipidemia by increasing anti-oxidant capacity and modulating iron deficiency [18]. These results therefore suggest that the mixed extract of *A. fistulosum* and *V. mandshurica*, which is rich in vitamins and essential minerals, can play an important role in the treatment of obesity and obesity-related disorders such as hyperlipidemia and insulin resistance.

We investigated the effect of AFE, VME, and the mixed extract of *A. fistulosum* and *V. mandshurica* (AFE + VME) in HFD-induced obese mice and found that AFE + VME effectively ameliorated obesity by synergistically reducing body weight, fat mass, and serum triglyceride and cholesterol levels. In addition, the mixed extract increased the mRNA levels of PPAR-γ, UCP-2, and adiponectin and decreased leptin expression in the epididymal white adipose tissue. UCP-2 is involved in the regulation of thermogenesis, energy expenditure, and obesity [19]. The adipocytes secrete various bioactive proteins called adipocytokines, such as leptin and adiponectin, into the blood circulation. Leptin, the gene product of the obesity gene, is directly associated with the regulation of adipose tissue mass and body weight in humans and rodents [20]. Adiponectin plays a major role in the modulation of glucose and lipid metabolism in insulin-sensitive tissues in both humans and animals [21]. PPAR-γ is a major ligand-activated transcription factor for regulating the various adipocyte genes and adipocyte differentiation [21]. Thiazolidinedione (TZD), a specific synthetic ligand activator of PPAR-γ, improves glucose tolerance, insulin sensitivity, and lipid metabolism in type 2 diabetes mellitus and obesity [22, 23]. The administration of TZDs has been shown to increase the expression and plasma concentration of adiponectin via the activation of the adiponectin promoter in diabetic patients and animals with insulin resistance [24]. Our results suggest that the mixed extract of *A. fistulosum* and *V. mandshurica* improved lipid metabolism via the up-regulation of PPAR-γ, UCP-2, and adiponectin expression and down-regulation of leptin in adipose tissue.

Previous phytochemical studies have revealed that the extract of *A. fistulosum* contains ferulic acid and quercetin [25] and the extract of *V. mandshurica* contains esculetin [26]. These phenolic compounds were shown to effectively inhibit adipocyte differentiation and reduce body weight, fat accumulation, and oxidation as well as inflammation in HFD-induced obese mice [27–29]. Although the anti-obesity effects of these constituents has not been demonstrated in this work, these results suggest that these phenolic compounds may synergistically contribute to the anti-obesity and hypolipidemic effects of the mixed extract of *A. fistulosum* and *V. mandshurica*.

## Conclusion

The oral administration of the mixed extract of *A. fistulosum* and *V. mandshurica* (AFE + VME) significantly reduced body weight, fat mass, and adipocyte size, and improved serum triglyceride, total cholesterol, and leptin levels in high-fat-diet-induced obese mice. The mixed extract also decreased the serum levels of AST and ALT, which are markers of liver injury. The anti-obesity and hypolipidemic effects of AFE + VME were associated with the up-regulation of the mRNA expression of PPAR-γ, UCP-2, and adiponectin and the down-regulation of leptin in high-fat-diet-induced obese mice. Therefore, AFE + VME may be a potentially effective therapy for obesity and its related metabolic disorders.

## Abbreviations

AFE: *Allium fistulosum* extract
ALT: Alanine aminotransferase
AST: Aspartate aminotransferase
FER: Food efficiency ratio
HDL: High-density lipoprotein
HFD: High fat diet
LDL: Low-density lipoprotein
ND: Normal diet
PPAR-γ: Peroxisome proliferator-activated receptor-γ
RDA: Recommended daily allowance
UCP-2: Uncoupling protein-2
VME: *Viola mandshurica* extract
